# Elevated β1-Adrenergic Receptor Autoantibody Levels Increase Atrial Fibrillation Susceptibility by Promoting Atrial Fibrosis

**DOI:** 10.3389/fphys.2020.00076

**Published:** 2020-02-12

**Authors:** Luxiang Shang, Ling Zhang, Mengjiao Shao, Min Feng, Jia Shi, Zhenyu Dong, Qilong Guo, Jiasuoer Xiaokereti, Ran Xiang, Huaxin Sun, Xianhui Zhou, Baopeng Tang

**Affiliations:** ^1^Department of Pacing and Electrophysiology, The First Affiliated Hospital of Xinjiang Medical University, Urumqi, China; ^2^Institute of Clinical Medical Research, The First Affiliated Hospital of Xinjiang Medical University, Urumqi, China

**Keywords:** atrial fibrillation, β1-adrenergic receptor autoantibody, atrial fibrosis, circulating fibrosis biomarker, autoimmune

## Abstract

**Objective:**

Beta 1-adrenergic receptor autoantibodies (β1ARAbs) have been identified as a pathogenic factor in atrial fibrillation (AF), but the underlying pathogenetic mechanism is not well understood. We assessed the hypothesis that elevated β1ARAb levels increase AF susceptibility by promoting atrial fibrosis.

**Methods:**

A total of 70 patients with paroxysmal AF were continuously recruited. The serum levels of β1ARAb and circulating fibrosis biomarkers were analyzed by ELISA. Linear regression was used to examine the correlations of β1ARAb levels with left atrial diameter (LAD) and circulating fibrosis biomarker levels. Furthermore, we established a rabbit β1ARAb overexpression model. We conducted electrophysiological studies and multielectrode array recordings to evaluate the atrial effective refractory period (AERP), AF inducibility and electrical conduction. AF was defined as irregular, rapid atrial beats > 500 bpm for > 1000 ms. Echocardiography, hematoxylin and eosin staining, Masson’s trichrome staining, and picrosirius red staining were performed to evaluate changes in atrial structure and detect fibrosis. Western blotting and PCR were used to detect alterations in the protein and mRNA expression of TGF-β1, collagen I and collagen III.

**Results:**

Patients with a LAD ≥ 40 mm had higher β1ARAb levels than patients with a smaller LAD (8.87 ± 3.16 vs. 6.75 ± 1.34 ng/mL, *P* = 0.005). β1ARAb levels were positively correlated with LAD and circulating biomarker levels (all *P* < 0.05). Compared with the control group, the rabbits in the immune group showed the following: (1) enhanced heart rate, shortened AERP (70.00 ± 5.49 vs. 96.46 ± 3.27 ms, *P* < 0.001), increased AF inducibility (55% vs. 0%, *P* < 0.001), decreased conduction velocity and increased conduction heterogeneity; (2) enlarged LAD and elevated systolic dysfunction; (3) significant fibrosis in the left atrium identified by Masson’s trichrome staining (15.17 ± 3.46 vs. 4.92 ± 1.72%, *P* < 0.001) and picrosirius red staining (16.76 ± 6.40 vs. 4.85 ± 0.40%, *P* < 0.001); and (4) increased expression levels of TGF-β1, collagen I and collagen III.

**Conclusion:**

Our clinical and experiential studies showed that β1ARAbs participate in the development of AF and that the potential mechanism is related to the promotion of atrial fibrosis.

## Introduction

Atrial fibrillation (AF), the most common clinical cardiac arrhythmia, has high morbidity, disability and mortality and has become a serious public health issue and socioeconomic burden worldwide (Chugh et al., 2014; Ribeiro and Otto, 2018). To date, the pathophysiological mechanisms of AF initiation and progression have not been completely elucidated. Emerging evidence indicates that autoimmunity mechanisms play an important role in the development of AF and might be a direct cause of and contributor to AF in some patients (Stavrakis et al., 2009; Lee et al., 2011; Gollob, 2013).

The beta 1-adrenergic receptor (β1AR) is a type of transmembrane receptor belonging to the cardiovascular G-protein-coupled receptor family. β1AR is the predominant β-adrenoceptor subtype in the human heart, accounting for 60–70% and 70–80% of β-adrenoceptors in the human atrium and ventricle, respectively (Wallukat, 2002). Early studies have confirmed that elevated levels of autoantibodies against β1AR (β1ARAbs) can induce cardiomyopathy and heart failure (Wallukat et al., 1991). Current evidence from both clinical studies and animal-model experiments has revealed that β1ARAb is also of pathogenic importance in AF. Yalcin et al. (2015a) found that serum β1ARAb levels were higher in patients with paroxysmal AF (PAF) than in healthy controls, and β1ARAb levels were an independent predictor of PAF occurrence. Furthermore, Hu et al. (2016) demonstrated an ascending gradient of serum β1ARAb levels from healthy control subjects to PAF patients to patients with persistent AF. Additionally, the area under the curve of β1ARAb concentration predicted AF recurrence after cryoablation in patients with PAF (Yalcin et al., 2015b). Evidence from experiments in a rabbit autoimmune model showed that animals developed high titers of β1ARAbs after immunization with the second extracellular loop (ECL2) peptide of β1AR, and elevated β1ARAbs reduced the atrial effective refractory period (AERP) and facilitated AF induction (Li et al., 2015, 2016). However, the underlying pathogenetic mechanisms of β1ARAb-mediated AF remains unclear.

Atrial fibrosis is one of the fundamental mechanisms of AF (Burstein and Nattel, 2008), and previous studies showed that β1ARAbs could induce cardiomyopathy by promoting ventricular fibrotic structural remodeling (Matsui et al., 1999; Giménez et al., 2005). Thus, we hypothesize that atrial fibrosis might be an important mechanism in β1ARAb-mediated AF. To this end, we measured the levels of β1ARAb in patients with PAF and analyzed their correlation with the presence of atrial fibrosis via non-invasive assessments that included echocardiographic LA diameter and circulating fibrosis biomarkers. Furthermore, we established a rabbit model by passive immunization against β1ARAbs; in this model, we examined whether increased expression of β1ARAbs plays a role in atrial fibrotic remodeling.

## Materials and Methods

### Ethics Committee Approval

The protocol for the clinical study was approved by the Medical Ethics Committee of the First Affiliated Hospital of Xinjiang Medical University (Approval Number: 20170213-02) and conformed to the principles of the Declaration of Helsinki. All participants provided written informed consent for participation. The protocol for the experimental study was approved by the Institutional Animal Care and Use Committee of the First Affiliated Hospital of Xinjiang Medical University (Approval Number: IACUC-20170420-03) and conformed to the principles of the International Association of Veterinary Editors’ Consensus Guidelines as well as the Basel Declaration. Anesthesia procedures were performed with pentobarbital sodium (30 mg/kg) via the marginal ear vein, and pentobarbital sodium was given as needed to maintain the depth of anesthesia during surgery. All efforts were made to minimize animal suffering.

### Study Population and Data Collection

From July 2017 to March 2018, 70 consecutive patients who were newly diagnosed with PAF and were admitted to the Heart Center of the First Affiliated Hospital of Xinjiang Medical University were recruited. PAF was defined as AF episodes cardioverted within 7 days after onset, as stated by the 2016 European Society of Cardiology AF guidelines (Kirchhof et al., 2016). Patients with autoimmune diseases, heart failure with reduced ejection fraction, history of acute coronary syndrome, severe valvular heart disease, or infectious diseases were excluded from our study. Participants underwent echocardiographic examination by experienced sonographers with a GE Vivid E9 ultrasound instrument (GE Vingmed Ultrasound, Horten, Norway). Information on demographic and clinical characteristics was obtained for all recruited patients.

### Enzyme-Linked Immunosorbent Assay (ELISA)

A 5 mL peripheral vein blood sample from each patient was collected in a non-anticoagulant blood-collection tube in the morning after a 12 h fast. The blood was centrifuged at 3000 rpm for 10 min to obtain the serum, which was then stored at -80°C until processing. The serum levels of β1ARAb, procollagen type III N-terminal peptide (PIIINP), procollagen type I C-terminal peptide (PICP), and galectin-3 (Gal3) were measured quantitatively using ELISA kits according to the manufacturer’s instructions. PIIINP, PICP and Gal3 were analyzed using kits produced by Elabscience (E-EL-H0183c, E-EL-H0196c, and E-EL-H1470c; Beijing, China), and the kit used to measure β1ARAbs was from Cusabio (CSB-E15079h; Wuhan, China).

### Experimental Animals and Design

**FIGURE 1:**
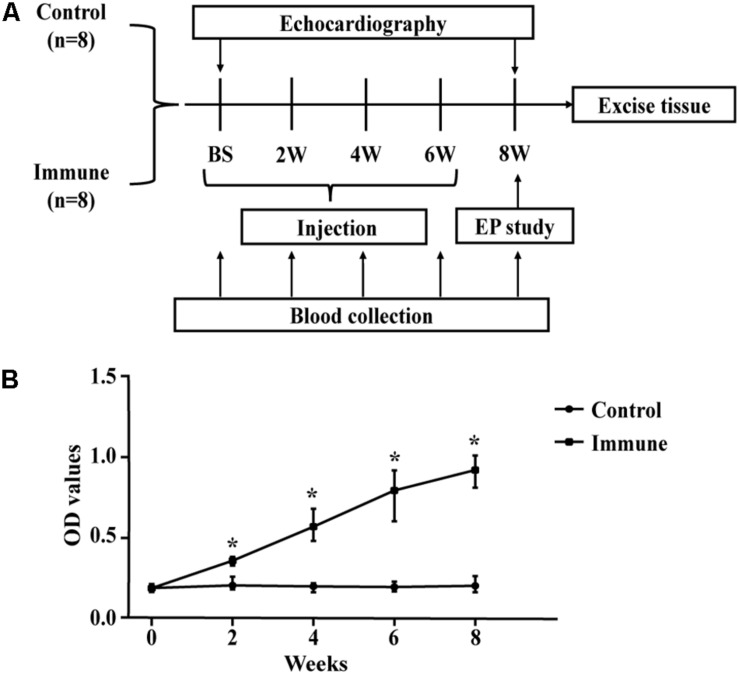
Schematic of the study process. **(A)** study protocol diagram; **(B)** ELISA results (β1ARAb OD values) in the two groups. *P < 0.05, control group vs. immune group at the same time point. ELISA, enzyme-linked immunosorbent assay; β1ARAb, beta 1-adrenergic receptor autoantibody; OD, optical density.

Sixteen male New Zealand white rabbits (each weighing 3.0–3.5 kg at baseline) were obtained from the Experimental Animal Center of Xinjiang Medical University (Urumqi, China) and were randomly divided into two groups: a control group and an immune group. The generation of the animal model followed the protocols of previous studies (Li et al., 2014, 2015, 2016), and a flow chart of the experimental design is shown in Figure 1A. The immune group was initially immunized with 2 mg of the β1AR ECL2 peptide (amino acid sequence: ^197^HWWRAESDEARRCYNDPKCCDFVTNR^223^; 98.13% purity, synthesized by Biosynthesis Biotechnology Inc., Beijing, China) in 1 mL of complete Freund’s adjuvant (Sigma-Aldrich, St. Louis, MO, United States) at 0 weeks and was boosted with 2 mg of the β1AR ECL2 peptide and incomplete Freund’s adjuvant (2 mg in 1 mL; Sigma-Aldrich, St. Louis, MO, United States) three times, spaced 2 week apart. Rabbits in the control group received an equal amount of adjuvant without the β1AR ECL2 peptide at the same time points. Blood samples were collected from both groups every 2 weeks in an awake state before injection, and the serum β1ARAb levels were measures by ELISA and expressed as optical density values based on previous studies (Li et al., 2015). Transthoracic echocardiographic examination was performed at baseline and repeated at 8 weeks after the first immunization. Atrial electrophysiological studies, multielectrode array (MEA) measurement and histological analyses were performed at 8 weeks.

### Echocardiography

Echocardiographic examinations of all rabbits were performed with a PHILIPS HD11XE transthoracic doppler ultrasound imaging system (Philips Inc., Bothell, WA, United States) with an S12-4 scan probe by an experienced sonographer who was blinded to the nature of the animal experiment. After the rabbits were anesthetized, the hair in the anterior chest area was shaved, the rabbits were placed in the left lateral decubitus position, and the measurements were taken. The left atrial diameter (LAD), right atrial diameter (RAD), left ventricular end-diastolic dimension (LVEDD), left ventricular end-systolic dimension (LVESD), right ventricular diameter (RVD), and left ventricular ejection fraction (LVEF) were measured. Each result was recorded as the average across three consecutive cardiac cycles.

### Electrophysiological Measurement

Under anesthesia, surface electrocardiogram (ECG) leads were placed onto the extremities of the animals and connected to a computer-based multichannel physiological laboratory system (LEAD-7000, Jinjiang Electronic Science and Technology Inc., Chengdu, China) to record the heart rate for 5 min. Spontaneous arrhythmia episodes within 5 min were also documented. Then, the neck region was shaved, iodine disinfectant was applied, and an incision was made. The right jugular vein was isolated and intubated with a 4F sheath. The quadripolar electrode catheter entered the right atrium under the control of ECG, and the atrial potential was recorded in combination with surface ECG.

AERP and AF inducibility were measured as described in our previous study (Wang et al., 2017). AERP was conducted with a programmed train of eight basic stimuli (S1-S1 = 260 ms) followed by one extra stimulus (S2) with an initial pacing length of 200 ms and 5 ms decrements until S2 failed to capture the depolarization. The longest S1-S2 interval was defined as the AERP. The AERP was measured three times, and the average value was calculated. The inducibility of AF was assessed by burst pacing to the right atrium (twofold threshold current, cycle length 50 ms, duration 30 s per bout), and this treatment was repeated 5 times for each rabbit. AF inducibility was calculated as the percentage of successfully recorded AF. AF was defined as irregular, rapid atrial beats > 500 beats per minute (bpm) for more than 1000 ms (Yang et al., 2018). Other types of arrhythmias induced in the rabbit were defined as follows: (1) atrial premature beat: a premature P wave that is morphologically a variant or replica of the P waves of the baseline rhythm; (2) sinus tachycardia: regular, rapid heart rate > 250 bpm with 1:1 atrioventricular conduction arising from the sinus node; and (3) atrial flutter: regular, rapid atrial beats > 250 bpm with distinct P waves between variable QRS cycles (Curtis et al., 2013).

### Flexible MEA Recording

MEA measurement was performed at sinus rhythm following electrophysiological study to record the conduction and conduction heterogeneity of the LA appendage epicardial surface *in vivo*. The chest of each rabbit was opened by cutting the center of the sternum, and the heart was exposed. A flexible MEA chip with 36 electrodes (6 × 6 electrodes, interelectrode distance: 300 μm, electrode diameter: 30 μm) was positioned at the surface of the LA appendage (Supplementary Figure S1). When the unipolar electrograms of the MEA recording were stable, recordings were taken to generate an activation map and calculate the conduction velocity (CV). The inhomogeneity index was calculated as a variation coefficient of CV (P_5__–__95_/P_50_) (Lammers et al., 1990). Data were collected at 10 kHz per channel and analyzed with Cardio2D + software (Multi Channel Systems, Reutlingen, Germany).

### Histological Collection and Processing

The animals were sacrificed with high doses of pentobarbital sodium at the end of the experiment, and the hearts were quickly removed. The left atrial tissues were divided into small pieces, fixed in paraformaldehyde for histological staining, frozen in liquid nitrogen, and stored at −80°C for protein and mRNA analysis.

Atrial tissues were fixed in 4% paraformaldehyde for 24 h and then embedded in paraffin. The atrial tissue was sliced into 5 μm-thick cross-sections to visualize the cell structure. The sections were deparaffinized and subjected to hematoxylin and eosin (H&E) staining, Masson’s trichrome staining, and picrosirius red staining for assessment of basic tissue structure and detection of fibrosis following the methods of our previous study (Wang et al., 2017). Digital photographs were taken under a Leica microscope (DM2500, Wetzlar, Germany). The distribution of collagen area was measured by two staining methods (Masson’s trichrome staining and picrosirius red staining). The histopathological sections were analyzed using Image-Pro Plus software (version 6.0, Media Cybernetics, United States). The collagen area was calculated as the area of positive collagen staining divided by the entire myocardial area (%).

### Western Blotting Analysis

Western blot analysis was performed to detect the expression levels of transforming growth factor-β1 (TGF-β1), collagen I and collagen III; the protocols were as previously described (Wang et al., 2017). Briefly, 40μg of protein was fractionated by 12% SDS-PAGE and then transferred onto PVDF membranes. The membranes were blocked with 5% non-fat milk for 2 h and then incubated with primary antibodies overnight at 4°C. After being washed, the membranes were incubated with horseradish peroxidase-conjugated secondary antibodies for 2 h. Finally, the membranes were visualized with chemiluminescence reagents (EMD Millipore, Billerica, MA, United States). ImageJ 1.41 Software (NIH, Bethesda, MD, United States) was used to analyze the density of the Western blotting bands. The primary antibodies were as follows: anti-TGF-β1 (1:1000; Abcam, Cambridge, MA, United States), anti-collagen I (1:1000; Abcam, Cambridge, MA, United States) and anti-collagen III (1:1000; Bioss, Beijing, China) antibodies. All protein expression levels were normalized to the GAPDH expression level (1:1000; Goodhere Biotech, Hangzhou, China).

### Real-Time Polymerase Chain Reaction (RT-PCR)

RT-PCR was used to quantitatively describe the mRNA expression of TGF-β1, collagen I and collagen III. Total RNA was extracted with TRIzol Reagent (Ambion, Austin, TX, United States) according to the manufacturer’s instructions. RT-PCR was performed on an ABI QuantStudio 6 Flex RT-PCR System (Applied Biosystems, United States) with the SYBR Green I incorporation method. The relative expression levels of mRNAs were calculated using the 2^–ΔΔ*Ct*^ method. GAPDH was used as the internal control. The primers for related genes are listed in Table 1.

**TABLE 1:** Primer sequences for RT-PCR.

Genes	Forward	Reverse
TGF-β1	5′-AGCTGTACATTGACTTCCGCAAGG-3′	5′-CAGGCAGAAGTTGGCGTGGTAG-3′
Collagen I	5′-AACTTGCCTTCATGCGTCTG-3′	5′-CCTCGGCAACAAGTTCAACA-3′
Collagen III	5′-CGGACTTGCAGGAATTACAGG-3′	5′-TTTCCGTCTCTTCCAGGTTCA-3′
GADPH	5′-CAGGGCTGCTTTTAACTCTGG-3′	5′-TGGAAGATGGTGATGGCCTT-3′

**RT-PCR, reverse transcription polymerase chain reaction; TGF-β1, transforming growth factor-beta 1; GAPDH, glyceraldehyde-3-phosphate dehydrogenase.**

### Statistical Analysis

Data analysis was performed using SPSS software (version 23.0, SPSS Inc., Chicago, IL, United States). Continuous data are presented as the means and standard deviations and were evaluated by Student’s *t*-test. Classification data were presented as proportions and evaluated by the chi-squared test or Fisher’s exact test. Correlations between β1ARAb levels and other parameters were ascertained by Pearson’s correlation coefficient or Spearman’s rank correlation coefficient. A two-tailed *P* < 0.05 was considered statistically significant.

## Results

### Clinical Characteristics of the Study Population

This study enrolled 70 patients with PAF; their baseline clinical characteristics are described in Table 2. According to LA anteroposterior diameter, participants were divided into two groups: a group with LA diameter < 40 mm (*n* = 47) and a group with LA diameter ≥ 40 mm (*n* = 23). Patients with atrial enlargement had a higher level of β1ARAbs (8.87 ± 3.16 vs. 6.75 ± 1.34 ng/mL, *P* = 0.005) and a lower LVEF (60.21 ± 4.87 vs. 62.95 ± 5.47%, *P* = 0.045) than those without atrial enlargement. No differences in age, sex, comorbidity, blood biochemical parameters or fibrosis-related biomarkers were observed between the two groups (all *P* > 0.05).

**TABLE 2:** The clinical characteristics of the study population.

Characteristics	Total participants (*n* = 70)	LA anteroposterior diameter		*P*-value
		<40 mm (*n* = 47)	≥40 mm (*n* = 23)	
Age, years	60.57 ± 13.25	60.17 ± 13.47	61.39 ± 13.04	0.720
Male, n (%)	39 (55.7)	27 (57.4)	12 (52.2)	0.677
Hypertension, n (%)	33 (47.1)	21 (44.7)	12 (52.2)	0.555
Diabetes mellitus, n (%)	15 (21.4)	10 (21.3)	5 (21.7)	0.965
Coronary heart disease, n (%)	12 (17.1)	8 (17.0)	4 (17.4)	0.969
History of stroke, n (%)	14 (20.0)	7 (14.9)	7 (30.4)	0.127
CHA_2_DS_2_-VASc score	2.19 ± 1.84	2.09 ± 1.78	2.39 ± 1.99	0.518
HAS-BLED score	1.01 ± 1.07	0.94 ± 0.99	1.17 ± 1.23	0.386
LVEF, %	62.05 ± 5.40	62.95 ± 5.47	60.21 ± 4.87	0.045
ALT, U/L	20.00 ± 5.46	19.75 ± 5.60	20.52 ± 5.25	0.583
FBG, mmol/L	5.13 ± 0.82	4.97 ± 0.59	5.44 ± 1.12	0.069
TG, mmol/L	1.59 ± 1.01	1.68 ± 1.14	1.39 ± 0.63	0.168
TC, mmol/L	3.76 ± 0.96	3.78 ± 0.88	3.73 ± 1.12	0.855
LDLC, mmol/L	2.35 ± 0.78	2.33 ± 0.76	2.40 ± 0.85	0.745
HDLC, mmol/L	1.11 ± 0.31	1.13 ± 0.33	1.09 ± 0.27	0.602
β1ARAbs, ng/mL	7.45 ± 2.33	6.75 ± 1.34	8.87 ± 3.16	0.005
PIIINP, ng/mL	17.09 ± 6.30	16.17 ± 4.72	18.96 ± 8.51	0.081
PICP, ng/mL	4.10 ± 0.81	4.03 ± 0.61	4.26 ± 1.11	0.254
Gal3, ng/mL	2.02 ± 0.34	1.96 ± 0.29	2.13 ± 0.40	0.058

**Values are presented as the mean ± SD, or n (%). LA, left atrial; LVEF, left ventricular ejection fraction; ALT, alanine transferase; FBG, fasting blood glucose; TG, triglyceride; TC, total cholesterol; LDLC, low-density lipoprotein cholesterol; HDLC, high-density lipoprotein cholesterol; β1ARAbs, beta 1-adrenergic receptor autoantibodies; PIIINP, procollagen type β N-terminal peptide; PICP, procollagen type C-terminal peptide; Gal3, galectin-3.**

### Correlation of β1ARAb Levels With Fibrosis and Clinical Indexes

**FIGURE 2:**
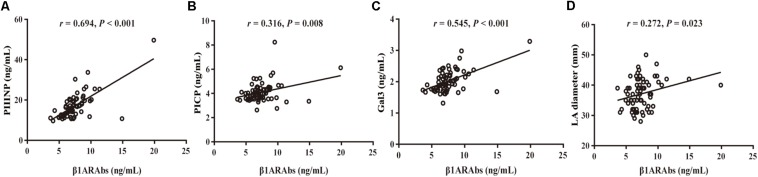
Correlation of serum β1ARAb levels with echocardiographic indexes and fibrosis-related biomarkers (**A**, PIIINP; **B**, PICP; **C**, Gal3; **D**, LA diameter). β1ARAb, beta 1-adrenergic receptor autoantibody; PIIINP, procollagen type III N-terminal peptide; PICP, procollagen type I C-terminal peptide; Gal3, galectin-3; LA, left atrial.

As demonstrated in Figure 2, Pearson’s linear correlation analysis showed that the serum β1ARAb levels were positively correlated with LA diameter and circulating biomarkers (LA diameter: *r* = 0.272, *P* = 0.023; PIIINP: *r* = 0.694, *P* < 0.001; PICP: *r* = 0.316, *P* = 0.008; Gal3: *r* = 0.545, *P* < 0.001). β1ARAb levels did not correlate significantly with other clinical indexes, including age, LVEF, hypertension, diabetes mellitus, coronary heart disease, history of stroke, CHA_2_DS_2_-VASc score and HAS-BLED score (Table 3).

**TABLE 3:** Correlation analysis between β1ARAb levels and clinical indexes in patients with PAF.

	*r*	*P*-value
Age	–0.009	0.939
LVEF	–0.108	0.374
Hypertension	–0.070	0.564
Diabetes mellitus	–0.187	0.121
Coronary heart disease	0.024	0.841
History of stroke	0.140	0.249
CHA_2_DS_2_-VASc	–0.140	0.248
HAS-BLED	–0.021	0.861

**β1ARAb, beta 1-adrenergic receptor autoantibody; LVEF, left ventricular ejection fraction.**

### Effect of β1ARAbs on Atrial Electrophysiology

All rabbits in both groups survived through the entire research period. From the second week onward, the rabbits in the immune group, immunized with the β1AR ECL2 peptide, developed higher levels of β1ARAb than the control group (Figure 1B).

**FIGURE 3:**
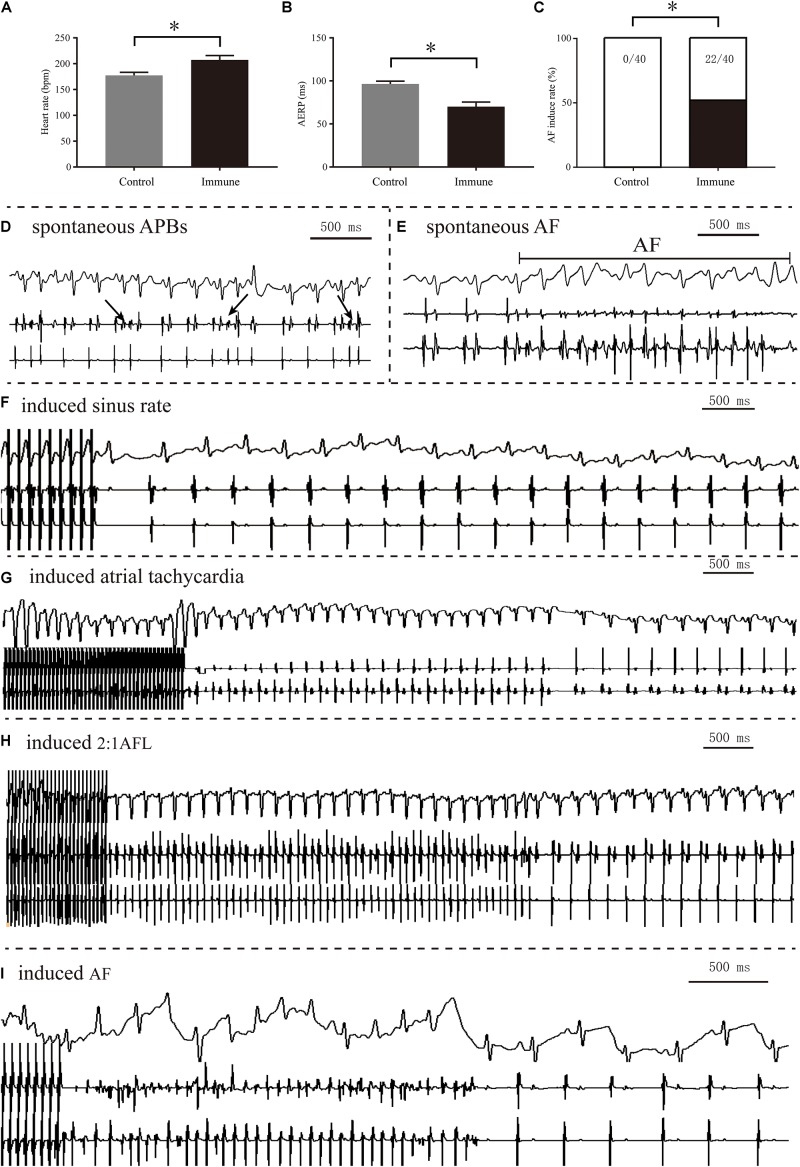
Electrophysiology measurements of the two groups. Comparison of heart rate **(A)**, AERP **(B)**, and AF inducibility **(C)** between the two groups; spontaneous APBs **(D)** and AF **(E)** observed in the immune group; representative sinus rhythm **(F)**, sinus tachycardia **(G)**, AFL episode **(H)** and AF episode **(I)** induced after burst pacing. *P < 0.05, control group vs. immune group. AERP, atrial effective refractory period; AF, atrial fibrillation; APB, atrial premature beat; AFL, atrial flutter.

Spontaneous AF episodes were observed in two rabbits in the immune group in the observational period prior to the invasive electrophysiological study, but the rate difference between groups was not statistically significant (2/8 vs. 0/8, *P* > 0.05). Compared with the control group, the immune group showed a significantly increased heart rate (207.13 ± 8.63 vs. 177.13 ± 6.17 bpm, *P* < 0.001), shortened AERP (70.00 ± 5.49 vs. 96.46 ± 3.27 ms, *P* < 0.001), and increased rate of induced AF (55% vs. 0%, *P* < 0.001) 8 weeks after immunization (Figure 3).

**FIGURE 4:**
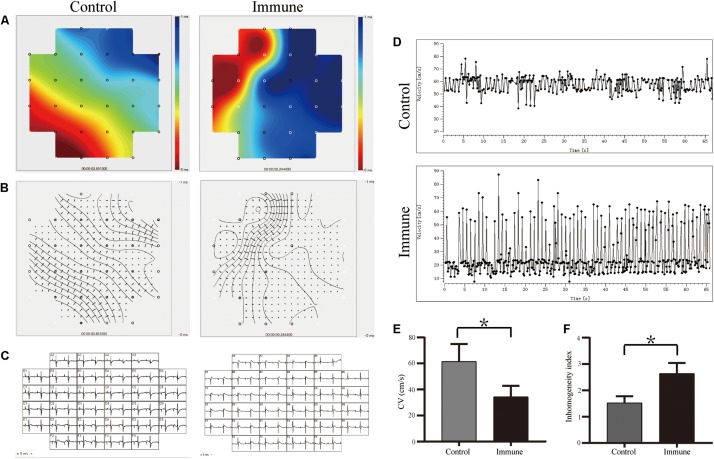
β1ARAbs reduced the LA conduction function. **(A)** Representative color maps of epicardial multielectrode activation in LA appendage; areas of isochronal crowding were found in rabbits of immune group; **(B)** representative examples of conduction heterogeneity map in LA appendage; **(C)** recording propagation map during sinus rhythm; **(D)** conduction maps of activation in LA appendage; β1ARAbs reduced CV **(E)** and increased conduction heterogeneity **(F)**. *P < 0.05, control group vs. immune group. β1ARAbs, beta 1-adrenergic receptor autoantibodies; LA, left atrial; CV, conduction velocity.

Figures 4A–C demonstrate the conduction activation maps and CV maps of the LA appendage epicardial surface; a slower CV (34.38 ± 8.48 vs. 61.50 ± 13.40 cm/s, *P* < 0.001, Figures 4D,E) and greater conduction inhomogeneity index (2.63 ± 0.40 vs. 1.52 ± 0.25, *P* < 0.001, Figures 4D,F) were observed in the immune group than in the control group.

### Changes in Echocardiography Parameters

**FIGURE 5:**
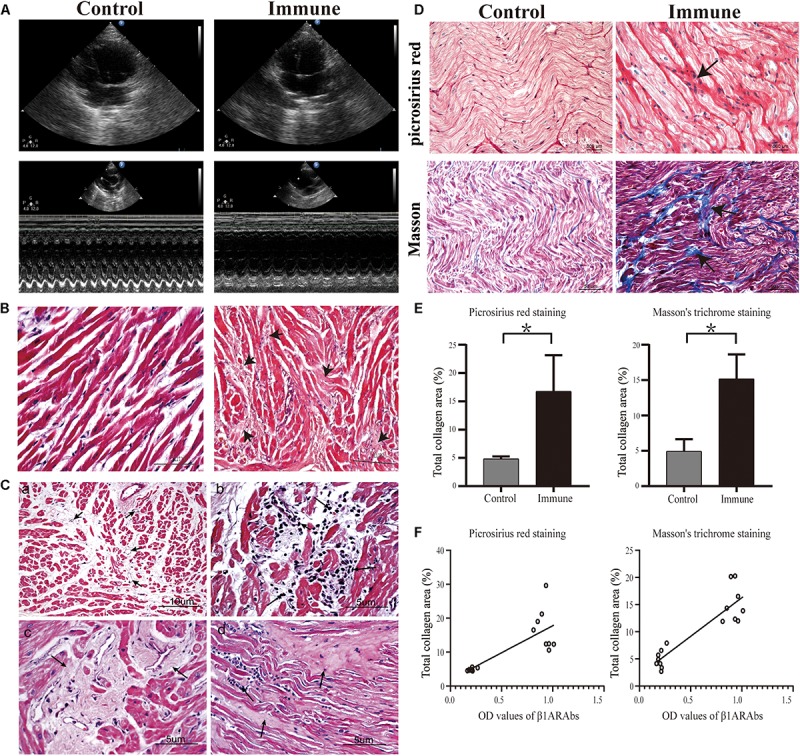
Schematic diagram of echocardiography results and histopathological changes in atrial tissues. **(A)** increased LAD and reduced cardiac function in the immune group; **(B)** representative images of H&E staining (40×); **(C)** angiogenesis (a, 20×), inflammatory cell infiltration (b, 40×), interstitial fibrosis (c, 40×) and increased ECM (d, 40×) in the left atrium of the immune group; **(D)** representative images of picrosirius red staining (40×) and Masson’s trichrome staining (20×); **(E)** quantitative assessment of atrial fibrosis between two groups; **(F)** correlation of β1ARAb OD values of 8 weeks with total collagen area. *P < 0.05, control group vs. immune group. LAD, left atrial diameter; H&E, hematoxylin and eosin; ECM, extracellular matrix; β1ARAb, beta 1-adrenergic receptor autoantibody; OD, optical density.

Table 4 and Figure 5A show echocardiographic changes. In the immune group, increases in LAD, RAD, LVEDD, and LVESD and a decrease in LVEF were observed compared with the baseline levels. Compared to the control group, the immune group had significant increases in LAD, LVEDD, and LVESD and a significant decrease in LVEF after 8 weeks. There were no significant differences in LAD, RAD, LVESD, RVD, or LVEF between the baseline and endpoint in the control group, although LVEDD slightly increased after 8 weeks.

**TABLE 4:** Echocardiographic differences between the two groups.

Parameters	Control group			Immune group		
	Baseline	Endpoint	*P-*value	Baseline	Endpoint	*P-*value
LAD, mm	9.53 ± 0.43	9.77 ± 0.82	0.211	9.13 ± 0.64	10.93 ± 0.99*	0.012
RAD, mm	9.63 ± 0.71	10.53 ± 1.22	0.081	9.40 ± 1.11	11.23 ± 0.96	0.004
LVEDD, mm	15.18 ± 0.83	16.05 ± 0.89	0.047	14.56 ± 0.97	16.89 ± 0.44*	0.001
LVESD, mm	10.67 ± 0.86	11.42 ± 1.08	0.158	9.99 ± 0.97	12.94 ± 1.02*	< 0.001
RVD, mm	8.33 ± 0.59	8.55 ± 0.62	0.153	8.16 ± 0.62	8.61 ± 0.34	0.054
LVEF, %	65.12 ± 5.14	63.71 ± 6.82	0.682	67.43 ± 5.77	54.47 ± 9.68*	0.001

*P < 0.05 compared with the control group at the same time point. LAD, left atrial diameter; RAD, right atrial diameter; LVEDD, left ventricular end-diastolic dimension; LVESD, left ventricular end-systolic dimension; RVD, right ventricular diameter; LVEF, left ventricular ejection fraction.

### Histopathological Changes

In the control group, H&E staining showed that the cardiomyocytes were arranged neatly, and there was a small amount of connective tissue in the extracellular matrix (ECM, Figure 5B). However, the interstitial structure was disordered in the immune group, with extensive fibrous tissue hyperplasia accompanied by inflammatory cell infiltration and increased angiogenesis (Figure 5C).

Additionally, increased collagen accumulation in the ECM in the immune group was observed by Masson’s trichrome staining (15.17 ± 3.46 vs. 4.92 ± 1.72%, immune and control groups, respectively, *P* < 0.001) and picrosirius red staining (16.76 ± 6.40 vs. 4.85 ± 0.40%, immune and control groups, respectively, *P* < 0.001, Figures 5D,E). Correlation analysis showed a significant positive correlation between circulating β1ARAb levels at 8 weeks and total fiber area (*r* = 0.895 and 0.786 for Masson’s trichrome staining and picrosirius red stain, respectively, both *P* < 0.001, Figure 5F).

### Fibrosis-Related Protein and Gene Expression

**FIGURE 6:**
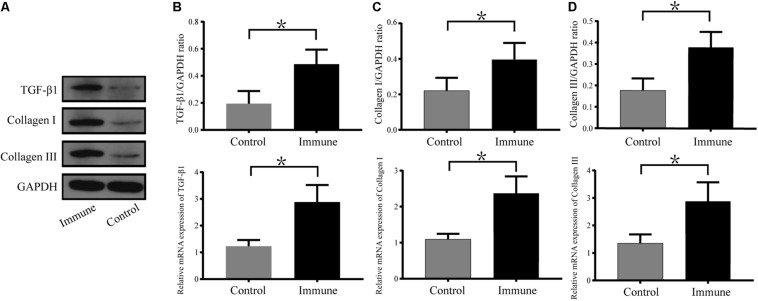
Expression levels of TGF-β1, collagen I and collagen III in atrial tissues. **(A)** Western blotting results for TGF-β1, collagen I and collagen III in the two groups. Relative protein and mRNA expression of TGF-β1 **(B)**, collagen I **(C)**, and collagen III **(D)** are shown, following normalization to GAPDH. *P < 0.05, control group vs. immune group. TGF-β1, transforming growth factor-β1; GAPDH, glyceraldehyde-3-phosphate dehydrogenase.

As shown in Figure 6, the messenger RNA and protein expression levels of TGF-β1, collagen I and collagen III were significantly upregulated in the immune group compared with the control group.

## Discussion

### Main Finding

In the present study, we analyzed the relationship between β1ARAbs and non-invasive atrial fibrosis indicators in patients with PAF and examined the effects of atrial structural remodeling in a rabbit model with enhanced β1ARAbs expression. The major findings were as follows: (1) β1ARAb levels were positively correlated with LA anterior-posterior diameter and three circulating fibrosis markers (PIIINP, PICP, Gal3) in PAF patients; (2) excessive expression of β1ARAbs increased LAD and interstitial fibrosis and led to increased inducibility of AF along with shortened AERP, slowed CV and increased conduction heterogeneity.

### β1ARAbs in AF Patients

Fibrosis is an important part of atrial remodeling in AF, and an increased LA diameter is a simple indicator of severe atrial structural remodeling and interstitial fibrosis or scarring (Liao et al., 2017). In addition, left atrium enlargement is the pathological basis and a major determinant of AF and its progression. Our study reported for the first time that β1ARAb levels have a positive linear correlation with LA diameter but are not associated with other clinical manifestations or comorbidities, suggesting that β1ARAbs may cause atrial structural remodeling and might be involved in the development of AF. This result supports the previous observation that patients with persistent AF have higher β1ARAb levels than patients with PAF (Hu et al., 2016). At the same time, our finding might also partly explain why elevated β1ARAb levels increased the AF recurrence rate after cryoablation in a previous study (Yalcin et al., 2015b), as enlarged LA size is a well-known, consistent, independent predictor of recurrence following ablation in AF; its predictive ability has been confirmed in large observational studies and meta-analyses (Zhuang et al., 2012; Jin et al., 2018). Furthermore, Duan et al. (2019) found that in hypertrophic cardiomyopathy patients, those with LA diameters ≥ 50 mm had significantly higher levels of β1ARAbs than those with smaller LA diameters [52.78 (46.76, 58.34) vs. 45.03 (36.74, 55.44) ng/mL, *P* = 0.042]. Our result was similar to that of a recently published study investigating the association of another G-protein-coupled receptor autoantibody (against the M2-muscarinic acetylcholine receptor) with atrial fibrosis in AF patients; a positive correlation was found between serum autoantibody levels and collagen volume in LA appendages (Ma et al., 2019).

The main feature of atrial fibrosis is increased collagen deposition in the ECM, and human atrial fibrosis is mainly composed of types I and III collagen (Boldt et al., 2004). In the process of atrial fibrosis development, components for collagen synthesis, secretion, renewal, and deposition, such as PICP and PIIINP, are released into the blood and can be used as circulating biomarkers (Dilaveris et al., 2019). Gal3, a member of the galectin family, is elevated in fibrotic conditions and highly expressed in fibrotic cardiac tissues (Calvier et al., 2013; Clementy et al., 2018). In our study, serum β1ARAb levels were correlated with the levels of three fibrosis-related biomarkers. To date, however, there have been conflicting results regarding the predictive value of circulating biomarkers in atrial fibrosis. Data from patients undergoing cardiac surgery demonstrated that serum PICP levels correlated significantly with the percentage of LA fibrosis from atrial biopsy specimens (Swartz et al., 2012). Nevertheless, other studies failed to verify the correlation between these biomarkers and fibrosis as assessed by LA low-voltage area (Begg et al., 2017). A convincing explanation is that the blood levels of fibrosis-related biomarkers are susceptible to non-cardiac fibrosis, and systemic fibrosis masks their peripheral levels (Begg et al., 2018). In our study, we pre-excluded patients with severe heart failure and those with autoimmune or infectious diseases; only patients newly diagnosed with PAF were included. We hoped that, once diseases which could cause non-atrial fibrosis were mainly eliminated, the levels of these biomarkers could indicate the severity of atrial fibrosis. In addition, our results showed only weak to moderate correlations between β1ARAb levels and non-invasive atrial fibrosis indexes, although the correlations were statistically significant. The clinical significance must be interpreted with caution.

### Elevated β1ARAbs Increase AF Susceptibility in an Animal Model

We observed spontaneous AF in two immunized rabbits, although the intergroup difference in proportion was not statistically significant (2/8 vs. 0/8). Nonetheless, this finding suggested that elevated β1ARAbs might be a direct cause of AF. We also evaluated the AF propensity of these animals, and the results showed that the heart rate was enhanced and AERP was shortened after immunization, which was similar to the results of a previous study (Li et al., 2015). However, we observed a much higher rate of induced AF (22/40) than reported in that previous study (1/24), probably because we used a higher dose of injected β1ARAbs and a longer modeling time in our study. These results suggest that β1ARAbs and AF may have a dose-response relationship, as previous studies have also shown a significant negative correlation between β1ARAb levels and AERP (Li et al., 2015).

### β1ARAbs Promote Atrial Structural Remodeling and Related Mechanisms

Previous studies have fully demonstrated that the long-term overexpression of β1ARAbs can cause ventricular structural changes and contribute to cardiomyopathy and heart failure progression (Jahns et al., 2004; Zuo et al., 2011). Our results were consistent with those of previous studies and generally corresponded to the expected results, with LVEDD and LVESD being increased and LVEF being decreased in the immune group after 8 weeks. In this present study, we focused on the structural changes in the atrium, and we found that overexpression of β1ARAbs led to atrial structural remodeling, manifested by increased LA diameter, heavy collagen deposition in the ECM, and increased protein and mRNA expression of collagen I and III, indicating that the heart damage caused by β1ARAbs includes both atrial and ventricular damage. This result was also consistent with the reduction in CV and the increase in conduction heterogeneity in the immune group on MEA recording, as extensive atrial fibrosis leads to disturbances in electrical conduction, and fibrous scars impede the normal conduction of atrial myocytes. Previous studies have verified that β1ARAbs specifically bind to β1ARs and exhibit agonistic activity against them, resulting in myocardial damage and cardiac dysfunction (Wang et al., 2019). Since β1AR is one of the predominant adrenergic receptors widely distributed throughout the myocardial tissue, this might explain why β1ARAbs also damage the atrium. Our results were similar to those of a previous study in an autoimmune myocarditis rat model, in which researchers found that the inducibility of AF was dependent on atrial structural remodeling rather than inflammation (Hoyano et al., 2010).

Our study did not delve into the mechanism-based signaling pathways that regulate β1ARAb-mediated atrial fibrosis. However, numerous studies have shown that at the cellular and molecular levels, various cardiac damage and pathogenic factors cause atrial fibrosis through the shared mechanism of fibroproliferative signaling, and TGF-β1 is a key mediator of ECM protein expression and fibrosis (Lijnen et al., 2000). In our models, the protein and mRNA expression levels of TGF-β1 were significantly elevated, indicating that the TGF-β1 signaling pathway is activated in the β1ARAb overexpression model. A previous study found that β1ARAbs activated the β1AR/cAMP/PKA pathway and promoted the proliferation and activation of cardiac fibroblasts (Lv et al., 2016). Activated cardiac fibroblasts are characterized by increased synthesis of collagen I and III and increased deposition of those proteins in the ECM (Cavin et al., 2014). In addition, many cytokines, such as IL-6 and TGF-β1, are secreted by fibroblasts in response to stimulation by pathogenic factors (Fu et al., 2012; Cavin et al., 2014). More interestingly, TGF-β1 can modulate cardiomyocyte survival and activate fibroblasts (Zhang et al., 2018). Therefore, we speculated that active cardiac fibroblasts and TGF-β1 could promote each other, forming a positive feedback loop that promotes β1ARAb-induced atrial fibrosis and AF propensity, but further verification is needed. In addition, previous research also confirmed that β1ARAb activated the canonical cAMP/PKA signaling pathway in cardiomyocytes, leading to functional alterations in intracellular calcium handling (Jane-wit et al., 2007). Sustained β1ARAb agonism eventually elicited caspase-3 activation and promoted cardiomyocyte apoptosis *in vivo* (Jane-wit et al., 2007).

### Therapeutic Implications of β1ARAb-Mediated AF

Evidence from clinical and experimental studies illustrates that immunoadsorption of circulating β1ARAbs improved cardiac function in cardiomyopathy (Matsui et al., 2006; Nagatomo et al., 2017). However, immunoadsorption therapy has the shortcomings of high cost, logistical challenges, and considerable time and labor requirements; oral drug treatment is increasingly coming to the fore (Jahns et al., 2006; Wess et al., 2019). Since β1ARAbs produce downstream effects by stimulating cardiac β1ARs, β-adrenoceptor inhibitors might be an effective treatment for β1ARAb-mediated injury. Evidence from an experimental study showed that nebivolol attenuates TGF-β1 pathways in a renovascular hypertension disease model (Ceron et al., 2013). Further studies are needed to determine whether β-adrenoceptor blockers have beneficial antifibrotic effects in a β1ARAb overexpression model.

### Limitations

Our study has several limitations that should be mentioned. First, the cross-sectional study design did not allow us to address the causal relationship between β1ARAb levels and atrial fibrosis in PAF patients, however, we compensated by performing animal experiments. Second, we did not apply more-accurate methods to evaluate the severity of atrial fibrosis, such as cardiac magnetic resonance imaging, electrophysiological mapping, or atrial tissue biopsy. Therefore, further larger-sample cohort studies of healthy controls and different types of AF patients are necessary to demonstrate the clinical significance of β1ARAbs in AF patients. Our experiment did not include groups with different concentrations of β1ARAbs, meaning that it could not address the dose-response relationship between β1ARAbs and AF. Numerous effectors and mechanisms, such as inflammatory reactions and cardiomyocyte apoptosis, are involved in the progression of cardiac fibrosis (Kong et al., 2014); whether these mechanisms are involved in β1ARAb-induced atrial fibrosis remains unclear and needs to be addressed in further research. Additionally, our study lacks pharmacological data; therefore, the results cannot be directly used in clinical practice.

## Conclusion

In conclusion, β1ARAb levels are positively correlated with LAD and circulating fibrosis-related biomarkers in patients with PAF. β1ARAb overexpression increases AF inducibility by facilitating atrial fibrosis, TGF-β1 signaling activation and collagen accumulation. Our results suggest that reversing atrial fibrosis may be a potential therapeutic target for the upstream prevention of β1ARAb-mediated AF.

## Data Availability Statement

The datasets generated for this study are available from the corresponding author on request.

## Ethics Statement

The studies involving human participants were reviewed and approved by the Medical Ethics Committee of the First Affiliated Hospital of Xinjiang Medical University. The patients provided their written informed consent to participate in this study. The animal study was reviewed and approved by the Institutional Animal Care and Use Committee of the First Affiliated Hospital of Xinjiang Medical University.

## Author Contributions

BT and XZ contributed to the funding acquisition, conception, and design of the study. LS, LZ, MS, MF, JS, ZD, and JX contributed to the animal experiments. QG, RX, and HS contributed to the clinical data collection and processing. LS, LZ, and MS contributed to the statistical analysis and interpretation. All authors contributed to the writing, critical reading, and approval of the manuscript.

## Conflict of Interest

The authors declare that the research was conducted in the absence of any commercial or financial relationships that could be construed as a potential conflict of interest.

## Supplementary Material

FIGURE S1 Schematic diagram of the flexible microelectrode array experimental setup and data acquisition.

## Abbreviations

AERP: atrial effective refractory period
AF: atrial fibrillation
β 1AR: beta 1-adrenergic receptor
β 1ARAb: beta 1-adrenergic receptor autoantibody
CV: conduction velocity
ECG: electrocardiogram
ECL2: second extracellular loop
ECM: extracellular matrix
ELISA: enzyme-linked immunosorbent assay
Gal3: galectin-3
H&E: hematoxylin and eosin
LA: left atrial
LAD: left atrial diameter
LVEDD: left ventricular end-diastolic dimension
LVEF: left ventricular ejection fraction
LVESD: left ventricular end-systolic dimension
MEA: multielectrode array
PAF: paroxysmal atrial fibrillation
PIIINP: procollagen type III N-terminal peptide
PICP: procollagen type I C-terminal peptide
RAD: right atrial diameter
RT-PCR: real-time polymerase chain reaction
RVD: right ventricular diameter
TGF- β 1: transforming growth factor- β 1.

## References

[B1] Begg G. A. Karim R. Oesterlein T. Graham L. N. Hogarth A. J. Page S. P. (2017). Intra-cardiac and peripheral levels of biochemical markers of fibrosis in patients undergoing catheter ablation for atrial fibrillation. *Europace* 19 1944–1950. 10.1093/europace/euw315 28339804

[B2] Begg G. A. Karim R. Oesterlein T. Graham L. N. Hogarth A. J. Page S. P. (2018). Left atrial voltage, circulating biomarkers of fibrosis, and atrial fibrillation ablation. *PLoS One* 13:e0189936. 10.1371/journal.pone.0189936 29293545 PMC5749720

[B3] Boldt A. Wetzel U. Lauschke J. Weigl J. Gummert J. Hindricks G. (2004). Fibrosis in left atrial tissue of patients with atrial fibrillation with and without underlying mitral valve disease. *Heart* 90 400–405. 10.1136/hrt.2003.015347 15020515 PMC1768173

[B4] Burstein B. Nattel S. (2008). Atrial fibrosis: mechanisms and clinical relevance in atrial fibrillation. *J. Am. Coll. Cardiol.* 51 802–809. 10.1016/j.jacc.2007.09.064 18294563

[B5] Calvier L. Miana M. Reboul P. Cachofeiro V. Martinez-Martinez E. de Boer R. A. (2013). Galectin-3 mediates aldosterone-induced vascular fibrosis. *Arterioscler. Thromb. Vasc. Biol.* 33 67–75. 10.1161/atvbaha.112.300569 23117656

[B6] Cavin S. Maric D. Diviani D. (2014). A-kinase anchoring protein-Lbc promotes pro-fibrotic signaling in cardiac fibroblasts. *Biochim. Biophys. Acta* 1843 335–345. 10.1016/j.bbamcr.2013.11.008 24269843

[B7] Ceron C. S. Rizzi E. Guimarães D. A. Martins-Oliveira A. Gerlach R. F. Tanus-Santos J. E. (2013). Nebivolol attenuates prooxidant and profibrotic mechanisms involving TGF-β and MMPs, and decreases vascular remodeling in renovascular hypertension. *Free Radic. Biol. Med.* 65 47–56. 10.1016/j.freeradbiomed.2013.06.033 23806385

[B8] Chugh S. S. Havmoeller R. Narayanan K. Singh D. Rienstra M. Benjamin E. J. (2014). Worldwide epidemiology of atrial fibrillation: a Global Burden of disease 2010 study. *Circulation* 129 837–847. 10.1161/circulationaha.113.005119 24345399 PMC4151302

[B9] Clementy N. Piver E. Bisson A. Andre C. Bernard A. Pierre B. (2018). Galectin-3 in atrial fibrillation: mechanisms and therapeutic implications. *Int. J. Mol. Sci.* 19:976. 10.3390/ijms19040976 29587379 PMC5979515

[B10] Curtis M. J. Hancox J. C. Farkas A. Wainwright C. L. Stables C. L. Saint D. A. (2013). The lambeth conventions (II): guidelines for the study of animal and human ventricular and supraventricular arrhythmias. *Pharmacol. Ther.* 139 213–248. 10.1016/j.pharmthera.2013.04.008 23588158

[B11] Dilaveris P. Antoniou C. K. Manolakou P. Tsiamis E. Gatzoulis K. Tousoulis D. (2019). Biomarkers associated with atrial fibrosis and remodeling. *Curr. Med. Chem.* 26 780–802. 10.2174/0929867324666170918122502 28925871

[B12] Duan X. Liu R. Luo X. Gao X. Hu F. Guo C. (2019). The relationship between β_1_-adrenergic, M_2_-muscarinic receptor autoantibody and hypertrophic cardiomyopathy. *Exp. Physiol.* 10.1113/EP088263 [Epub ahead of print]. 31808213

[B13] Fu Y. Xiao H. Zhang Y. (2012). Beta-adrenoceptor signaling pathways mediate cardiac pathological remodeling. *Front. Biosci. (Elite Ed.)* 4:1625–1637. 10.2741/e484 22201979

[B14] Giménez L. E. Hernández C. C. Mattos E. C. Brandão I. T. Olivieri B. Campelo R. P. (2005). DNA immunizations with M2 muscarinic and beta1 adrenergic receptor coding plasmids impair cardiac function in mice. *J. Mol. Cell. Cardiol.* 38 703–714. 10.1016/j.yjmcc.2004.12.009 15850564

[B15] Gollob M. H. (2013). Atrial fibrillation as an autoimmune disease? *Heart Rhythm* 10 442–443. 10.1016/j.hrthm.2013.01.023 23333723

[B16] Hoyano M. Ito M. Kimura S. Tanaka K. Okamura K. Komura S. (2010). Inducibility of atrial fibrillation depends not on inflammation but on atrial structural remodeling in rat experimental autoimmune myocarditis. *Cardiovasc. Pathol.* 19 e149–e157. 10.1016/j.carpath.2009.07.002 19747850

[B17] Hu B. Sun Y. Li S. Sun J. Liu T. Wu Z. (2016). Association of β1-adrenergic, M2-muscarinic receptor autoantibody with occurrence and development of nonvalvular atrial fibrillation. *Pacing Clin. Electrophysiol.* 39 1379–1387. 10.1111/pace.12976 27862036

[B18] Jahns R. Boivin V. Hein L. Triebel S. Angermann C. E. Ertl G. (2004). Direct evidence for a beta 1-adrenergic receptor-directed autoimmune attack as a cause of idiopathic dilated cardiomyopathy. *J. Clin. Invest.* 113 1419–1429. 10.1172/jci20149 15146239 PMC406525

[B19] Jahns R. Jahns V. Lohse Martin J. Palm D. (2006). Means for the inhibition of anti-ss1-adrenergic receptor antibodies. Patent No. WO2006EP02977. Würzburg: Julius-Maximilians-Universität 10.1172/jci200420149

[B20] Jane-wit D. Altuntas C. Z. Johnson J. M. Yong S. Wickley P. J. Clark P. (2007). Beta 1-adrenergic receptor autoantibodies mediate dilated cardiomyopathy by agonistically inducing cardiomyocyte apoptosis. *Circulation* 116 399–410. 10.1161/CIRCULATIONAHA.106.683193 17620508

[B21] Jin X. Pan J. Wu H. Xu D. (2018). Are left ventricular ejection fraction and left atrial diameter related to atrial fibrillation recurrence after catheter ablation? A meta-analysis. *Medicine (Baltimore)* 97:e10822. 10.1097/md.0000000000010822 29768386 PMC5976293

[B22] Kirchhof P. Benussi S. Kotecha D. Ahlsson A. Atar D. Casadei B. (2016). 2016 ESC guidelines for the management of atrial fibrillation developed in collaboration with EACTS. *Europace* 18 1609–1678. 27567465 10.1093/europace/euw295

[B23] Kong P. Christia P. Frangogiannis N. G. (2014). The pathogenesis of cardiac fibrosis. *Cell. Mol. Life Sci.* 71 549–574. 10.1007/s00018-013-1349-6 23649149 PMC3769482

[B24] Lammers W. J. Schalij M. J. Kirchhof C. J. Allessie M. A. (1990). Quantification of spatial inhomogeneity in conduction and initiation of reentrant atrial arrhythmias. *Am. J. Physiol.* 259(4 Pt 2) H1254–H1263. 1699438 10.1152/ajpheart.1990.259.4.H1254

[B25] Lee H. C. Huang K. T. Wang X. L. Shen W. K. (2011). Autoantibodies and cardiac arrhythmias. *Heart Rhythm* 8 1788–1795. 10.1016/j.hrthm.2011.06.032 21740882 PMC3855646

[B26] Li H. Murphy T. Zhang L. Huang B. Veitla V. Scherlag B. J. (2016). β1-adrenergic and M2 muscarinic autoantibodies and thyroid hormone facilitate induction of atrial fibrillation in male rabbits. *Endocrinology* 157 16–22. 10.1210/en.2015-1655 26517045

[B27] Li H. Scherlag B. J. Kem D. C. Benbrook A. Zhang L. Huang B. (2014). Atrial tachyarrhythmias induced by the combined effects of β1/2-adrenergic autoantibodies and thyroid hormone in the rabbit. *J. Cardiovasc. Transl. Res.* 7 581–589. 10.1007/s12265-014-9573-5 24903978 PMC5811990

[B28] Li H. Zhang L. Huang B. Veitla V. Scherlag B. J. Cunningham M. W. (2015). A peptidomimetic inhibitor suppresses the inducibility of β1-adrenergic autoantibody-mediated cardiac arrhythmias in the rabbit. *J. Interv. Card. Electrophysiol.* 44 205–212. 10.1007/s10840-015-0063-8 26446828 PMC5812008

[B29] Liao Y. C. Liao J. N. Lo L. W. Lin Y. J. Chang S. L. Hu Y. F. (2017). Left atrial size and left ventricular end-systolic dimension predict the progression of paroxysmal atrial fibrillation after catheter ablation. *J. Cardiovasc. Electrophysiol.* 28 23–30. 10.1111/jce.13115 27779351

[B30] Lijnen P. J. Petrov V. V. Fagard R. H. (2000). Induction of cardiac fibrosis by transforming growth factor-beta(1). *Mol. Genet. Metab.* 71 418–435. 10.1006/mgme.2000.3032 11001836

[B31] Lv T. Du Y. Cao N. Zhang S. Gong Y. Bai Y. (2016). Proliferation in cardiac fibroblasts induced by β1-adrenoceptor autoantibody and the underlying mechanisms. *Sci. Rep.* 6:32430. 10.1038/srep32430 27577254 PMC5006240

[B32] Ma G. Wu X. Zeng L. Jin J. Liu X. Zhang J. (2019). Association of autoantibodies against M2-muscarinic acetylcholine receptor with atrial fibrosis in atrial fibrillation patients. *Cardiol. Res. Pract.* 2019:8271871. 10.1155/2019/8271871 30863630 PMC6378765

[B33] Matsui S. Fu M. L. Hayase M. Katsuda S. Yamaguchi N. Teraoka K. (1999). Active immunization of combined beta1-adrenoceptor and M2-muscarinic receptor peptides induces cardiac hypertrophy in rabbits. *J. Card. Fail.* 5 246–254. 10.1016/s1071-9164(99)90009-x 10496197

[B34] Matsui S. Larsson L. Hayase M. Katsuda S. Teraoka K. Kurihara T. (2006). Specific removal of beta1-adrenoceptor autoantibodies by immunoabsorption in rabbits with autoimmune cardiomyopathy improved cardiac structure and function. *J. Mol. Cell. Cardiol.* 41 78–85. 10.1016/j.yjmcc.2006.04.016 16780870

[B35] Nagatomo Y. McNamara D. M. Alexis J. D. Cooper L. T. Dec G. W. Pauly D. F. (2017). Myocardial recovery in patients with systolic heart failure and autoantibodies against β-adrenergic receptors. *J. Am. Coll. Cardiol.* 69 968–977. 10.1016/j.jacc.2016.11.067 28231950 PMC5330212

[B36] Ribeiro A. L. Otto C. M. (2018). Heartbeat: the worldwide burden of atrial fibrillation. *Heart* 104 1987–1988. 10.1136/heartjnl-2018-314443 30482808

[B37] Stavrakis S. Yu X. Patterson E. Huang S. Hamlett S. R. Chalmers L. (2009). Activating autoantibodies to the beta-1 adrenergic and m2 muscarinic receptors facilitate atrial fibrillation in patients with Graves’ hyperthyroidism. *J. Am. Coll. Cardiol.* 54 1309–1316. 10.1016/j.jacc.2009.07.015 19778674 PMC2801559

[B38] Swartz M. F. Fink G. W. Sarwar M. F. Hicks G. L. Yu Y. Hu R. (2012). Elevated pre-operative serum peptides for collagen I and III synthesis result in post-surgical atrial fibrillation. *J. Am. Coll. Cardiol.* 60 1799–1806. 10.1016/j.jacc.2012.06.048 23040566 PMC3482337

[B39] Wallukat G. (2002). The beta-adrenergic receptors. *Herz* 27 683–690. 10.1007/s00059-002-2434-z 12439640

[B40] Wallukat G. Morwinski M. Kowal K. Förster A. Boewer V. Wollenberger A. (1991). Autoantibodies against the beta-adrenergic receptor in human myocarditis and dilated cardiomyopathy: beta-adrenergic agonism without desensitization. *Eur. Heart J.* 12(Suppl. D) 178–181. 10.1093/eurheartj/12.suppl_d.178 1717272

[B41] Wang H. L. Zhou X. H. Li Z. Q. Fan P. Zhou Q. N. Li Y. D. (2017). Prevention of atrial fibrillation by using sarcoplasmic reticulum calcium atpase pump overexpression in a rabbit model of rapid atrial pacing. *Med. Sci. Monit.* 23 3952–3960. 10.12659/msm.904824 28811460 PMC5569926

[B42] Wang X. Zhang Y. Zhang J. Wang Y. X. Xu X. R. Wang H. (2019). Multiple autoantibodies against cardiovascular receptors as biomarkers in hypertensive heart disease. *Cardiology* 142 47–55. 10.1159/000497189 30982037

[B43] Wess G. Wallukat G. Fritscher A. Becker N. P. Wenzel K. Müller J. (2019). Doberman pinschers present autoimmunity associated with functional autoantibodies: a model to study the autoimmune background of human dilated cardiomyopathy. *PLoS One* 14:e0214263. 10.1371/journal.pone.0214263 31276517 PMC6611557

[B44] Yalcin M. U. Gurses K. M. Kocyigit D. Kesikli S. A. Ates A. H. Evranos B. (2015a). Elevated M2-muscarinic and β1-adrenergic receptor autoantibody levels are associated with paroxysmal atrial fibrillation. *Clin. Res. Cardiol.* 104 226–233. 10.1007/s00392-014-0776-1 25351416

[B45] Yalcin M. U. Gurses K. M. Kocyigit D. Kesikli S. A. Dural M. Evranos B. (2015b). Cardiac autoantibody levels predict recurrence following cryoballoon-based pulmonary vein isolation in paroxysmal atrial fibrillation patients. *J. Cardiovasc. Electrophysiol.* 26 615–621. 10.1111/jce.12665 25788224

[B46] Yang Y. Zhao J. Qiu J. Li J. Liang X. Zhang Z. (2018). Xanthine oxidase inhibitor allopurinol prevents oxidative stress-mediated atrial remodeling in alloxan-induced diabetes mellitus rabbits. *J. Am. Heart Assoc.* 7:e008807. 10.1161/jaha.118.008807 29720500 PMC6015332

[B47] Zhang Y. Lu Y. Ong’achwa M. J. Ge L. Qian Y. Chen L. (2018). Resveratrol inhibits the TGF-1-induced proliferation of cardiac fibroblasts and collagen secretion by downregulating miR-17 in rat. *Biomed. Res. Int.* 2018:8730593. 10.1155/2018/8730593 30648109 PMC6311767

[B48] Zhuang J. Wang Y. Tang K. Li X. Peng W. Liang C. (2012). Association between left atrial size and atrial fibrillation recurrence after single circumferential pulmonary vein isolation: a systematic review and meta-analysis of observational studies. *Europace* 14 638–645. 10.1093/europace/eur364 22117033

[B49] Zuo L. Bao H. Tian J. Wang X. Zhang S. He Z. (2011). Long-term active immunization with a synthetic peptide corresponding to the second extracellular loop of β1-adrenoceptor induces both morphological and functional cardiomyopathic changes in rats. *Int. J. Cardiol.* 149 89–94. 10.1016/j.ijcard.2009.12.023 20096470

