# Supplementary figures and images for: Elevated β1-Adrenergic Receptor Autoantibody Levels Increase Atrial Fibrillation Susceptibility by Promoting Atrial Fibrosis
Source: Front Physiol. 2020 Feb 12;11:76. doi: 10.3389/fphys.2020.00076 (PMC7028693; doi:10.3389/fphys.2020.00076)

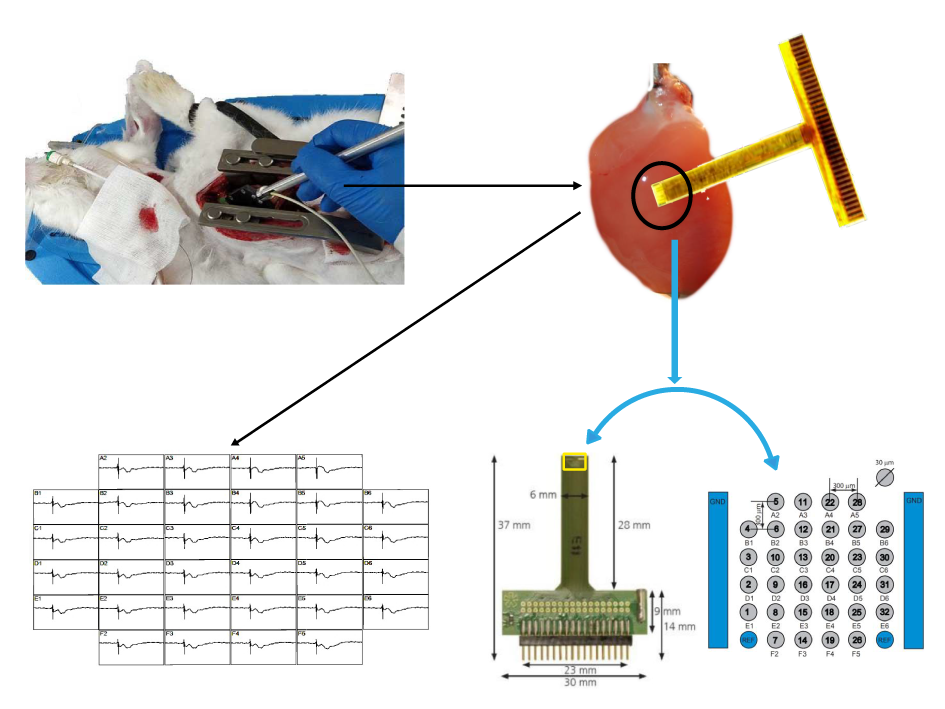

Supplement: FIGURE S1 — Schematic diagram of the flexible microelectrode array experimental setup and data acquisition. [file Image_1.TIF]
